# Editorial: New frameworks for chronic diseases treatment: research, prevention, intervention

**DOI:** 10.3389/fnbeh.2024.1540781

**Published:** 2025-01-07

**Authors:** Mauro Cozzolino, Giovanna Celia, Chiara Fioretti, Francesca Tessitore

**Affiliations:** ^1^Department of Humanities, Phylosophy and Education, University of Salerno, Fisciano, Italy; ^2^Department of Human Sciences, Education and Sport, University of Pegaso, Naples, Italy

**Keywords:** chronic disease, multidimensionality, clinical intervention, prevention, clinical research

According to epidemiological studies, chronic diseases, such as coronary heart disease, stroke, diabetes, cancer, genetic diseases, mental illnesses etc., are constantly increasing, not only in Western countries but also in developing countries.

Scientific literature on chronic diseases agrees on recognizing these as the result of a multifactorial combination of biological, cognitive, affective, behavioral and environmental components. An extensive body of research, in fact, linked chronic disease to processes and experiences occurring during intrauterine life, childhood or adult life, supporting the hypothesis of a cumulative exposure to stressful events and the evidence of experience-dependent gene expression (Bagby et al., 2019; Cozzolino and Celia, 2021; Cozzolino et al., 2017; Obrador et al., 2017; Renz et al., 2011). These findings represent an important example of how the stressful and/or traumatic experiences affect biological systems through epigenetic modulation, how to say all those modifications that can vary the phenotype of an individual, without however altering the genotype (Cozzolino et al., 2021; Cozzolino and Celia, 2021). This evidence also demonstrates the importance of a multidisciplinary approach in treating patients affected by chronic diseases (Cozzolino et al., 2021, 2017; Fioretti et al., 2024, 2020; Fioretti and Mugnaini, 2022).

The present Research Topic aimed at reflecting on the advances in prevention as well as in intervention for chronic diseases, enhancing a comprehensive overview of these conditions, with a particular emphasis on “multidimensionality,” intended as the capability to combine different levels of analysis (biochemical, cognitive, affective, environmental etc.), as key issue to address the multifactorial nature of chronic diseases. It aimed, therefore, to shed light on tasks, challenges and new perspectives of research and intervention with different kind of chronic diseases and patients, to understand the new contributions and possible directions for research and intervention in this field.

This Research Topic grouped a total of 4 articles that addressed the investigated topic from different perspectives. Even focusing on different kind of chronic diseases, all the papers share a multidimensional approach to the investigation on these, focusing on the implications both for prevention and intervention paths.

Cincidda et al. presented a pilot study aimed at proposing a new personalized psycho-social approach for supporting patients affected by prostate cancer (PC) and their caregivers' dyads. The authors well described how cancer disease represents a “family disease” and how the investigation of patients' as well as caregivers' psychological reactions to cancer diagnosis and characteristics of decision-making processes about the treatments becomes even more important to reach a multidimensional understanding of this condition, deepening the knowledge of its effects on the whole family system but also eventually enhancing the process of care. Specifically, the authors investigated the psychological impact of a newly diagnosed PC on patients' and caregivers' dyads and their alignment in the decision-making. The results showed low concordance in decision-making styles and preferences in patients and caregivers. Through the interpretations of their results, the authors stressed the importance of involving both patients and caregivers in decision making, suggesting the need to plan specific interventions for both patients and dyads, taking in consideration the mutual influence they have when dealing with a cancer diagnosis.

Fernandes et al., specifically focusing on stroke, carried out a scoping review to investigate the motivational strategies used by health care professionals in stroke survivors suggesting how the motivation plays a crucial role in increasing the limited adherence to rehabilitation programs for stroke survivors. Through their scoping review, the authors evidenced a wide range of interventions used by healthcare professionals to motivate stroke survivors that were grouped into a total of 11 strategies. The authors stressed the significance of engaging the patients but also their family in the rehabilitation process taking into consideration the importance of persuasion, encouragement and emotional support as pivotal motivation strategies. They also indicated a growing trend in incorporating new technologies to improve stroke survivors' adherence to rehabilitation programs. Their study offers interesting implication from a practical but also theoretical perspective since equipped the professionals with a toolkit to tailor patient-centered interventions aimed at enhancing the adherence to treatment and contributed to the ongoing debate on patient engagement in medical treatment.

He et al. deepened the investigation of osteoarthritis (OA) etiology confirming the relationship with systemic inflammatory response index (SIRI) levels. Their study represents the first large-scale study carried out on this Research Topic since they used cross-sectional data from the National Health and Nutrition Examination Survey (NHANES) database from 2005 to 2018. Their results indicated that SIRI measures the level of systemic inflammation in patients with OA and represents a potent predictor of disease activity, joint damage, and radiographic progression. In fact, the authors' results suggested that higher SIRI levels are significantly associated with increased risk of OA, highlighting the potential for using SIRI as a biomarker for early detection of OA. Their investigation had important implications because might enable healthcare practitioners to identify individuals at higher risk for developing OA and implement preventive measures. Overall, the study supported the need for anti-inflammatory therapies as part of the treatment protocol for OA patients. Therapies targeting systemic inflammation could be, therefore, highly beneficial in managing OA symptoms and improving patient outcomes.

Liu et al. performed a cross-sectional study investigating the consistency between self-reported disease diagnosis and clinical assessment of eight major chronic conditions. The general aims of the study were to explore the under-reporting of clinical conditions, assess potential factors associated with it, provide explanatory hypotheses to enhance health evaluation and public health decision-making processes. Using self-report questionnaires administered to 2,272 participants, the authors evaluated the prevalence of eight specific chronic conditions among the study participants, correlating the data with the results of physical examinations and laboratory tests, while investigating under-reporting, over-reporting, or agreement between them. The study results highlight a prevalence of under-reporting in four out of the eight chronic conditions, with advanced age and high BMI of the participants playing a predictive role. The study fosters an interesting discussion on the challenges of reporting chronic conditions by the population, highlighting the need to identify and target high-risk populations to provide support and organize preventive public health interventions.

## References

[B1] Bagby S. P. Martin D. Chung S. T. Rajapakse N. (2019). From the outside in: biological mechanisms linking social and environmental exposures to chronic disease and to health disparities. Am. J. Public Health 109, S56–S63. 10.2105/AJPH.2018.304864 30699032 PMC6356139

[B2] Cozzolino M. Celia G. (2021). The psychosocial genomics paradigm of hypnosis and mind–body integrated psychotherapy: experimental evidence. Am. J. Clin. Hypnosis 64, 123–138. 10.1080/00029157.2021.1947767 34723776

[B3] Cozzolino M. Cocco S. Piezzo M. Celia G. Costantini S. Abate V.. (2021). A psychosocial genomics pilot study in oncology for verifying clinical, inflammatory and psychological effects of mind-body transformations-therapy (MBT-T) in breast cancer patients: preliminary results. J. Clin. Med. 10:136. 10.3390/jcm10010136 33401546 PMC7796278

[B4] Cozzolino M. Guarino F. Castiglione S. Cicatelli A. (2017). Pilot study on epigenetic response to a mind-body treatment. Transl. Med. UniSa 17, 37–41. 30050879 PMC6056253

[B5] Fioretti C. Coppola S. Boscaino S. Celia G. Vastola R. Cozzolino M.. (2024). The effectiveness of Dragon Boat racing on body image and traumatic symptoms of breast cancer patients. Health Psychol. Res. 12:120055. 10.52965/001c.120055 38915786 PMC11196124

[B6] Fioretti C. Magni E. Barlocco F. Tomberli A. Baldini K. Ingles J.. (2020). Doctor-patient care relationship in genetic cardiomyopathies: an exploratory study on clinical consultations. PLoS ONE 15:e0236814. 10.1371/journal.pone.0236814 32756572 PMC7406074

[B7] Fioretti C. Mugnaini C. (2022). Living with type 1 diabetes mellitus in emerging adulthood: a qualitative study. Br. J. Health Psychol. 27, 1226–1240. 10.1111/bjhp.12596 35587032

[B8] Obrador G. T. Schultheiss U. T. Kretzler M. Langham R. G. Nangaku M. Pecoits-Filho R.. (2017). Genetic and environmental risk factors for chronic kidney disease. Kidney Int. Suppl. 7, 88–106. 10.1016/j.kisu.2017.07.004 30675423 PMC6341015

[B9] Renz H. Von Mutius E. Brandtzaeg P. Cookson W. O. Autenrieth I. B. Haller D.. (2011). Gene-environment interactions in chronic inflammatory disease. Nat. Immunol. 12, 273–277. 10.1038/ni0411-273 21423219

